# Intracellular Iron Chelation Modulates the Macrophage Iron Phenotype with Consequences on Tumor Progression

**DOI:** 10.1371/journal.pone.0166164

**Published:** 2016-11-02

**Authors:** Christina Mertens, Eman Abureida Akam, Claudia Rehwald, Bernhard Brüne, Elisa Tomat, Michaela Jung

**Affiliations:** 1 Institute of Biochemistry I, Goethe-University Frankfurt, Theodor-Stern-Kai 7, 60590 Frankfurt am Main, Germany; 2 University of Arizona, Department of Chemistry and Biochemistry, 1306 E., University Blvd., Tucson, AZ 85721-0041, United States of America; University of Nebraska Medical Center, UNITED STATES

## Abstract

A growing body of evidence suggests that macrophage polarization dictates the expression of iron-regulated genes. Polarization towards iron sequestration depletes the microenvironment, whereby extracellular pathogen growth is limited and inflammation is fostered. In contrast, iron release contributes to cell proliferation, which is important for tissue regeneration. Moreover, macrophages constitute a major component of the infiltrates in most solid tumors. Considering the pivotal role of macrophages for iron homeostasis and their presence in association with poor clinical prognosis in tumors, we approached the possibility to target macrophages with intracellular iron chelators. Analyzing the expression of iron-regulated genes at mRNA and protein level in primary human macrophages, we found that the iron-release phenotype is a characteristic of polarized macrophages that, in turn, stimulate tumor cell growth and progression. The application of the intracellular iron chelator (TC3-S_)2_ shifted the macrophage phenotype from iron release towards sequestration, as determined by the iron-gene profile and atomic absorption spectroscopy (AAS). Moreover, whereas the addition of macrophage supernatants to tumor cells induced tumor growth and metastatic behavior, the supernatant of chelator-treated macrophages reversed this effect. Iron chelators demonstrated potent anti-neoplastic properties in a number of cancers, both in cell culture and in clinical trials. Our results suggest that iron chelation could affect not only cancer cells but also the tumor microenvironment by altering the iron-release phenotype of tumor-associated macrophages (TAMs). The study of iron chelators in conjunction with the effect of TAMs on tumor growth could lead to an improved understanding of the role of iron in cancer biology and to novel therapeutic avenues for iron chelation approaches.

## Introduction

It is widely recognized that macrophages (MΦ) constitute one of the major cell populations infiltrating human tumors. High MΦ numbers are associated with poor outcome and correlate with tumor cell survival, neovascularization, and metastasis [1]. Tumor cell-derived factors skew MΦ towards a tumor-supporting phenotype in order to facilitate tumor growth and metastatic spread. MΦ exhibit a remarkable heterogeneity [1,2] and functional plasticity that can be described by two extreme phenotypes, known as M1- and M2-polarized MΦ [1,2]. M1-MΦ present the pro-inflammatory or “classically” activated phenotype, whereas M2-MΦ correlate to the anti-inflammatory or “alternatively” activated phenotype. However, these categories largely oversimplify the plasticity of MΦ, which presents a continuum of functional activation states that are determined by the local environment [1]. M1-MΦ mostly emerge during infectious or acute inflammatory conditions and are involved in combating pathogens, promoting the inflammatory response, and activating the adaptive immune response [2]. Anti-inflammatory M2-MΦ are mainly involved in the resolution of inflammation and regeneration [3–5]. Notably, the M2-like MΦ signature is also a characteristic of the tumor-associated macrophage (TAM) phenotype supporting tumor growth.

Polarization of MΦ is not only dependent on tumor cell-secreted mediators, but also on the iron content of the tissue. The availability of iron impacts the production and secretion of cytokines, thereby modulating the MΦ phenotype [6, 7]. MΦ are central in regulating iron homeostasis and represent the major source of available iron in the body. In response to inflammation, iron is sequestered in MΦ. This elicits a state of systemic inflammation-associated anemia, whereby the microenvironment is depleted and extracellular pathogen growth is limited [8–10]. In contrast, iron release by MΦ contributes to cell proliferation [11], which is important for tissue repair. In tumor tissue, MΦ are re-programmed to favor the survival of the growing tumor. Therefore, it might be speculated that tumor cells hijack the physiological role of MΦ in iron homeostasis as a source of iron within the tumor to ensure their own survival and growth. Considering the pivotal role of macrophages for iron homeostasis and the fact that the presence of macrophages was associated with histological grade and clinical prognosis in a variety of tumors [12], we sought to investigate the notion that TAMs could be a hitherto unrecognized target of antiproliferative iron chelators.

Current research typically focuses on studying the role of iron and iron-chelation therapy in tumor cells, whereas detailed knowledge on the crosstalk between tumor cells and TAMs as a possible source of iron is still lacking. Cancer cells require increased intracellular iron concentration in order to fulfill their enhanced metabolic turnover [13]. Therefore, cancer cells evolved a variety of mechanisms for enhanced uptake and storage of iron. Furthermore, a dysfunction or dysregulation in systemic iron homeostasis is often manifested in cancer patients due to chronic anemia, which is detected in approximately 80% of cancer patients [14]. Moreover, the expression of different iron-regulated genes such as transferrin receptor (TfR) [15], ferritin light (FTL) [16], and the iron regulatory protein (IRP)-2 [17] in tumor cells was correlated with a poor prognosis and a higher tumor grade, leading to increased chemoresistance. The role of iron in cancer progression was also documented by experimental approaches in animal models [18–20]. For instance, mice fed with low-iron diet prior to the implantation of tumor cells significantly delayed tumor growth [21]. Additionally, it was shown by the staining of iron deposits that the tumor outcompetes the natural iron reservoirs in liver and spleen [22]. Importantly, the expression levels of iron gene markers, particularly the iron exporter ferroportin, can be employed as independent predictors of prognosis in breast cancer [23] and might find important applications in individualized tumor therapy.

Given the apparent association of iron availability and tumorigenesis, a growing body of evidence anticipates the use of iron chelators as antineoplastic agents. Natural and synthetic iron chelators, such as Desferrioxamine, Deferiprone, and Deferasirox, have been utilized for several decades for the clinical treatment of iron overload due to chronic blood transfusion therapy (typically associated with genetic conditions such as β-thalassemia and sickle-cell anemia) [24]. More recently, iron chelators have been subjected to preclinical and clinical trials in order to evaluate their antineoplastic potential [25]. Despite promising effects of chelation therapy in experimental tumor models, low efficacy or narrow therapeutic windows have been reported in several studies of iron chelating agents in humans. These observations are attributed, at least in part, to the fact that current clinical chelators bind primarily labile plasma iron (also known as non-transferrin-bound iron, NTBI) and are not designed to target intracellular iron in malignant cells. Additionally, it was shown that the high hydrophilicity and unfavorable pharmacokinetic of common iron chelators such as Deferoxamine (DFO) make it difficult to reach an effective intratumoral concentration without unacceptable side effects [26]. The molecular design of new intracellular chelators, as well as prochelation strategies imparting selectivity towards malignant cells, are expected to enhance the scope of iron chelators as antiproliferative agents. Furthermore, a full understanding of the effects of iron chelation on all aspects of tumor growth is critical to the development of novel therapeutic strategies. While most of the current research was focused on tumor cells, the ability to inhibit potential sources of iron within the tumor microenvironment, such as TAMs, has not been explored.

Given the central function of MΦ in iron homeostasis and their crucial role during tumor progression, the current study aimed at defining the effects of the intracellular iron chelator (TC3-S_)2_ on the MΦ phenotype, especially in terms of their iron handling ability and functional effects with respect to cancer cells.

## Materials and Methods

### Materials

The disulfide prochelator (TC3-S)_2_ was synthesized as previously reported [27, 28]. Stock solutions were prepared at a standard concentration of 100 mM in dimethyl sulfoxide (DMSO). Solutions were always prepared freshly in degassed DMSO. Lipopolysaccharide (LPS) (0.5μg/ml) was purchased from Sigma-Aldrich (Steinheim, Germany), recombinant Interferon (IFN)γ (100U/ml) was obtained from Roche (Indianapolis, US),Interleukin (IL)-10 (20ng/ml) and IL-4 (20 ng/ml) from PeproTech (Hamburg, Germany), DMOG (1 mM) was delivered by Biomol (Hamburg, Germany), and Staurosporine (0.5 μg/ml) came from LC Laboratories (Woburn, USA).

### Stability of (TC3-S)_2_ in growth medium

UV-visible absorption spectra were recorded on an Agilent 8453 spectrophotometer. The aqueous stability of the disulfide prochelator (TC3-S)_2_ was determined using UV-visible absorption spectroscopy in full Eagle's Minimum Essential Medium (EMEM) growth medium without phenol-red. A stock solution of (TC3-S)_2_ was prepared in DMSO and diluted in complete growth medium for a final concentration of 12.0μM. The UV-Vis spectra were collected periodically for 24 hours while the temperature was maintained at 37.0°C. Concentrations of (TC3-S)_2_, at various times were determined using absorbance values at 318 nm relative to that at time zero.

### Primary macrophage generation

Human monocytes were isolated from commercially available, anonymized buffy coats obtained from a blood bank (DRK-Blutspendedienst Baden-Württemberg-Hessen, Frankfurt, Germany) using Ficoll-Hypaque gradients (PAA Laboratories, Cölbe, Germany). Peripheral blood mononuclear cells were washed twice with phosphate-buffered saline (PBS) containing 2 mM EDTA and subsequently incubated for 1 h at 37°C in RPMI 1640 medium supplemented with 100 U/ml penicillin and 100 μg/ml streptomycin to allow their adherence to culture dishes (Sarstedt, Nümbrecht, Germany). Non-adherent cells were removed. Monocytes were then differentiated into primary human MΦ with RPMI 1640 containing 5% AB-positive human serum (DRK-Blutspendedienst Baden-Württemberg-Hessen, Frankfurt, Germany) for 7 days and achieved approximately 80% confluence. For 24 h prior to stimulation cells were serum starved. Macrophages were then stimulated for different time periods (8–24 h) in RPMI medium with a combination of 0.5 μg/ml LPS and 100U/ml IFNγ (for M1 polarization) or 20 ng/ml IL-10 (for M2 polarization), alone or in combination with the chelator (100μM, last 6 h). The conditioned media of polarized macrophages were collected, centrifuged at 1000 xg for 5min., and aliquots were stored at -80°C until further use. Conditioned media was used for proliferation measurements in tumor cells. Supernatant of unstimulated macrophages served as control.

### MCF-7 cell culture

The human MCF-7 breast cancer cell line was purchased from ATCC-LGC Standard GmbH (Wesel, Germany). MCF-7 cells, were maintained in Dulbecco´s Modified Eagle medium (DMEM) with high glucose (Life Technologies, Darmstadt, Germany), supplemented with 100U/ml penicillin (PAA Laboratories, Cölbe, Germany), 100 μg/ml streptomycin (PAA Laboratories) and 10% heat-inactivated fetal calf serum (FCS; PAA Laboratories). Cells were kept in a humidified atmosphere with 5% CO_2_ at 37°C and passaged 3 times per week. For 24 hours prior to stimulation with macrophage-conditioned medium, cells were serum starved.

### RNA extraction and quantitative real-time PCR (qRT-PCR)

MΦ were stimulated either with LPS (0.5 μg/ml/IFNγ (100 U/ml) or IL-10 (20 ng/ml) for 8 h. Where indicated, IL-10 stimulation was supplemented with the chelator (TC3-S)_2_ at a concentration of 100 μM for the last 4 h of incubation. RNA was extracted using peqGold RNA Pure reagent (Peqlab Biotechnology, Erlangen, Germany). Total RNA (1μg) was transcribed using the Maxima first-strand cDNA synthesis kit (Fermentas, St. Leon-Rot, Germany). Quantitative real-time PCR was performed using the MyiQ real-time PCR system (Bio-Rad Laboratories) and Absolute Blue qPCR SYBR green fluorescein mix (Thermo Scientific, Karlsruhe, Germany). Real-time PCR results were quantified using the Bio-Rad CFX Manager (version 3.1) software program from Bio-Rad (Munich, Germany), with 18S mRNA expression as an internal housekeeping gene control for human samples. Sequences of primers were used as follows: 18S (NM_022551.2): sense, 5’-GTA-ACC-CGT-TGA-ACC-CCA-TT-3’, antisense, 5’-CCA-TCC-AAT-CGG-TAG-TAG-CG-3’, FPN(NM_014585.5): sense, 5´- TGA-GCC-TCC-CAA-ACC-GCT-TCC-ATA-3´, antisense, 5´-GGG-CAA-AAA-GAC-TAC-AAC-GAC-GAC-T-3´, FTL (NM_000146.3): sense, 5´-AGC-CTT-CTT-TGT-GCG-GTC-GGG-TA-3´, antisense, 5´- ACG-CCT-TCC-AGA-GCC-ACA-TCA-T-3´,HAMP (NM_021175.2): sense, 5´-TTT-TCG-GCG-CCA-CCA-CCT-TCT-T-3´, antisense, 5´-TTG-AGC-TTG-CTC-TGG-TGT-CTG-GGA-3´, CP (NM_000096.3): sense, 5´-CTT-TCC-TGC-TAC-CCT-GTT-TGA-TGC-3´, antisense, 5´-CTT-GCA-AAC-CGG-CTT-TCA-GA-3´, IRP2 (NM_004136.3): sense, 5´-GAA-ATA-TGG-TTC-AGG-AAA-CTC-CA-3´, antisense, 5´-GCC-AAA-ACA-GCT-TTC-ACA-CC-3´, TfR (NM_001128148.1): sense, 5´-ATG-GAA-TAA-AGG-GAC-GCG-GG-3´, antisense, 5´-TTA-TCA-GGG-ACA-GCC-AGA-CAC-AGC-3´, PCNA (NM_002592.2):sense, 5´-AAC-TCC-CAG-AAA-AGC-AAC-AAG-CA-3´, antisense, 5´-CGA-GGA-GGA-ATG-AGA-AGA-AGA-CG-3, Ki-67 (NM_002417.4): sense, 5´-CTT-TGG-GTG-CGA-CTT-GAC-G-3´, 5´-antisense, GTC-GAC-CCC-GCT-CCT-TTT-3´, TNFα (NM_000594.3): sense, 5’-GAC-AAG-CCT-GTA-GCC-CAT-GT-3’, antisense 5’-GAG-GTA-CAG-GCC-CTC-TGA-TG-3’, CD163 (NM_004244.5): sense, 5’-ACA-GCG-GCT-TGC-AGT-TTC-CTC-A-3’, antisense, 5’-GGC-TCA-GAA-TGG-CCT-CCT-TTT-CCA-3’, CCL18 (NM_002988.3): sense, 5’-CCC-AGC-TCA-CTC-TGA-CCA-CT-3’, antisense, 5’-GTG-GAA-TCT-GCC-AGG-AGG-TA-3’, CCL2 (NM_002982.3): sense, 5’-CAG-CCA-GAT-GCA-ATC-AAT-GCC-3’, antisense, 5’-TGG-AAT-CCT-GAA-CCC-ACT-TCT-3’, TGM2 (NM_004613.3): sense, 5’-GGC-ACC-AAG-TAC-CTG-CTC-A-3’, antisense, 5’-AGA-GGA-TGC-AAA-GAG-GAA-CG-3’.

### Western Blot analysis

For HIF-1α and PCNA Western analysis, cells were resuspended in lysis buffer containing 6.65 M urea, 10% glycerol, 1% SDS, and 10 mM Tris-HCl (pH 7.4). After sonification and centrifugation (15.000 xg, 5 min), the protein content was determined by the Lowry method according to manufacturer’s instructions (Bio-Rad, Munich, Germany). 100 μg protein was boiled in loading buffer, supplemented with 20% glycerol and bromphenol blue, loaded on a SDS gel and blotted using Immobilion-FL polyvinylidene difluoride (PVDF) membranes (Merck Millipore, Schwalbach, Germany). The membranes were blocked with 5% milk powder in TBST and analyzed using a specific antibody against HIF-1α in a 1:1000 (mouse, polyclonal; BD Bioscience, Heidelberg, Germany, 610959), for PCNA analysis a polyclonal antibody was used at a 1:200 dilution (rabbit, polyclonal, Santa Cruz, Heidelberg, Germany sc-7909). An antibody against nucleolin was used in a 1:3000 (rabbit, polyclonal, Santa Cruz, Heidelberg, Germany sc-13057) as loading control. For quantification, the fluorescence intensity was normalized to tubulin and is given relative to unstimulated controls. PCNA and nucleolin bands were visualized using the Odyssey infrared imaging system (Li-COR Biosciences GmbH, Bad Homburg, Germany).

For HIF-1α detection, secondary antibodies conjugated with horseradish peroxidase, following detection using enhanced chemiluminescence (ECL solutions from GE Healthcare, Little Chalfont, UK) were used.

### Flow cytometry

MΦ were stimulated either with LPS (0.5 μg/ml/IFNγ (100 U/ml), IL-10 (20 ng/ml) for 8 h in case of FTL and for 24 h for FPN detection. Where indicated, IL-10 stimulation was supplemented with the chelator (TC3-S)_2_ at a concentration of 100 μM for the last 4 h of incubation. MΦ were detached using Accutase (PAA) at 37°C and were transferred to FACS tubes for further treatment. To discriminate viable cells from apoptotic and necrotic cells, samples were stained with annexin V/propidium iodide (Annexin antibody and PI were purchased from Immunotools, Friesoythe, Germany), measured on a LSR Fortessa flow cytometer (BD Bioscience, Heidelberg, Germany), and analyzed using the FACS Diva system (BD Bioscience, San Jose, USA). As a positive control, cells were incubated with 0.5 μg/mL staurosporine (Sts; Sigma) for 4 hours at 37°C. FPN and FTL expression were analyzed using specific antibodies to recognize FPN (rabbit, polyclonal, Novus, Littleton, USA, NBP1-21502) and FTL (rabbit, monoclonal, abcam, Cambridge, UK, Ab109373) in a 1:100 dilution. Samples were measured and analyzed as described above. Polarization markers CD86 (mouse, monoclonal, BD Biosciences, clone 2331 (FUN-1), 555657), CD206 (mouse, monoclonal, BioLegend, San Diego, USA, clone 15–2, 321108), CD80 (mouse, monoclonal, BioLegend, clone 2D10, 305220), and CD163 (mouse, monoclonal, BD Bioscience, clone GHI/61, 556018) were measured and analyzed as described above.

### Proliferation assay

Proliferation of MCF-7 breast cancer cells was measured using the RTCA DP xCELLigence instrument (OLS, Bremen, Germany). Initially, a background measurement of the detector containing an E-plate insert was performed using 50 μl serum-free media incubated for 30 min in the incubator (37°C, 5% CO_2_). In parallel, human MCF-7 breast cancer cells were treated with trypsin, quantified, and added to the insert in 100 μl serum-free media (40.000 cells per well). MΦ-conditioned media were added. Proliferation was measured as an increase in impedance continuously for a period of 99 h. Data are presented as the slope per hour of the normalized cell index as a measure for the time-dependent changes in impedance. The RTCA Software 1.2 (OLS, Bremen, Germany) was used for both data acquisition and analysis. For the determination of the proliferation markers PCNA and Ki-67, MCF-7 cells were treated for 24 h with MΦ-conditioned media and measured by qRT-PCR as described above.

### Adhesion assay

MCF-7 cells were stimulated with MΦ-conditioned media for 2 days and labelled with cell tracker green (Life Technologies). 5 x 10^4^ MCF-7 cells were seeded on collagen I (10 μg/ml; BD Biosciences) or fibronectin (10 μg/ml; Sigma Aldrich) pre-coated wells for 2 h, washed, and fixed with 4% PFA. 5 pictures were taken from each group from at least three independent experiments using triplicates and the number of attached cells was quantified.

### Migration

Migration of MCF-7 breast cancer cells was measured using the RTCA DP xCELLigence instrument. Initially, the bottom of the upper chamber was pre-coated with collagen I (10 μg/ml; BD Biosciences). Afterwards, a background measurement of the detector containing a two-chamber CIM-plate insert was performed using 30 μl serum-free media incubated for 30 min in the incubator (37°C, 5% CO_2_). In parallel, human MCF-7 breast cancer cells were treated with trypsin, quantified, and added to the upper chamber in 100 μl MΦ-conditioned media (40.000 cells per well). After 30 min incubation at RT, migration was measured as the increase in impedance continuously for a period of 4 h. Data are presented as the slope as a measure for the time-dependent changes in impedance. The RTCA Software 1.2 was used for both data acquisition and analysis.

### Atomic absorption spectrometry

MΦ were stimulated either with LPS (0.5 μg/ml/IFNγ (100 U/ml) or IL-10 (20 ng/ml) for 24 h. Where indicated, IL-10 stimulation was supplemented with the chelators (TC3-S)_2_ at a concentration of 100 μM for the last 4 h of incubation. The iron content of MΦ supernatants was determined by graphite furnace atomic absorption spectrometry. Samples were measured as triplicates with a PinAAcle^™^ 900 T Atomic Absorption Spectrometer (PerkinElmer, Waltham, USA). Slit 0.2 nm and wavelength 248,33 nm were used as spectrometer parameters. A hollow cathode iron lamp (30 mA maximum operating current) was run at 100% maximum current. The calibration solutions (10 μg/l to 90 μg/l) were prepared by adequate dilution of Iron Standard for AAS (Sigma-Aldrich, Steinheim, Germany) stock solution. A pyrolysis temperature of 1400°C and an atomization temperature of 2100°C were used.

### Statistical analysis

Each experiment was performed at least three times. The p values were calculated using One-way ANOVA and Tukey’s post-hoc test considered to be significant at *p<0.05, **p<0.01, ***p<0.001.

## Results

### Polarization dictates the macrophage iron phenotype

**Fig 1:**
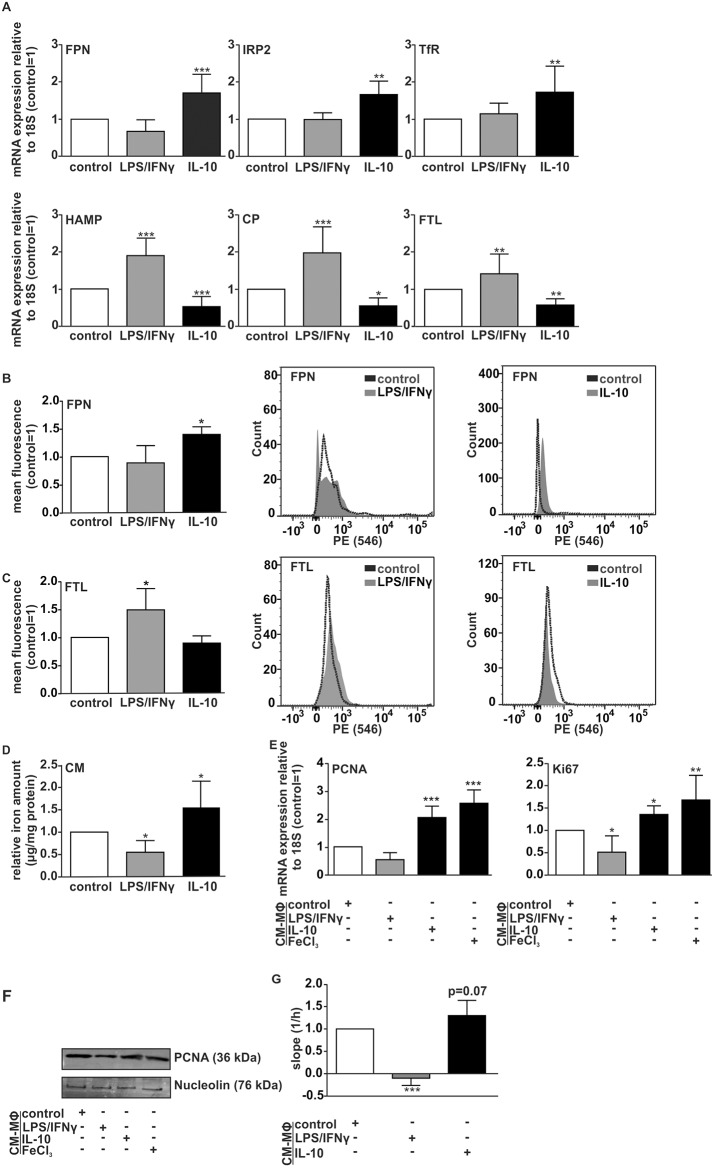
Polarization dictates the macrophage iron phenotype. (A) mRNA expression of iron regulated genes FPN, IRP2, TfR, HAMP, CP, and FTL was quantified in primary human macrophages using qRT-PCR. Results were normalized to the unstimulated control. Protein expression of FPN (B) and FTL (C) was determined in macrophages by FACS analysis. (D) Iron content in macrophage supernatants was quantified by AAS measurement. (E) MCF-7 breast cancer cells were stimulated with macrophage conditioned media (CM) and proliferation was analyzed by qRT-PCR of (E) PCNA, Ki-67, (F) Western analysis of PCNA, and (G) xCELLigence. Data are means ± S.D.M, n>6, *p<0.05, **p<0.01, ***p<0.001 vs. control/CM_control.

In order to test the functionality of intracellular iron chelation on macrophage iron polarization, we first established an experimental cell culture set-up that allows us to modify the macrophage iron phenotype. Considering previous findings from Recalcati et al. [11], primary human MΦ were either stimulated with a combination of LPS/IFNγ in order to induce a classically activated M1-phenotype or polarized towards M2 using IL-10. The purity of the M2 culture was confirmed by analyzing polarization markers at protein (S1A Fig) and mRNA level (S1B Fig), observing a significant up-regulation of anti-inflammatory markers and a decrease of pro-inflammatory mediators. We then analyzed the iron-regulated gene profile at mRNA level by qRT-PCR measurements (Fig 1A) and at protein level by flow cytometry (Fig 1B and 1C). The iron exporter FPN is decreased in LPS/IFNγ-treated MΦ, both at mRNA and protein level. In contrast, M2-polarization upon IL-10 stimulation significantly up-regulates FPN mRNA and protein levels. In line with these findings, we observed a significant up-regulation of its natural ligand HAMP by stimulation with LPS/IFNγ and a significant IL-10-induced decrease. Furthermore, we analyzed the expression of IRP2 and TfR. Both genes remained unaffected by LPS/IFNγ-stimulation, but were significantly induced upon IL-10-stimulation. Moreover, Ceruloplasmin (CP), a protein endowed with ferroxidase activity, was significantly induced by LPS/IFNγ and showed a decreased expression after IL-10-treatment. The iron storage protein FTL showed a significantly increased mRNA expression upon LPS/IFNγ treatment, which was confirmed at protein level. On the contrary, FTL was down-regulated in IL-10-stimulated MΦ, both at mRNA and protein level. The results obtained for the iron-release phenotype could be reproduced and confirmed by polarization of primary human macrophages with IL-4 (S2 Fig), which was in line with previous work [6].

As a functional readout, we quantified the iron content in MΦ supernatants (CM) by atomic-absorption-spectrometry (AAS) (Fig 1D). Analysis of the iron content showed a significant increase of iron in the supernatant of IL-10-stimulated M2-polarizedMΦ, whereas a substantial decrease was observed for LPS/IFNγ treated M1-MΦ compared to unstimulated control.

In order to define the functional consequences of MΦ-polarization in terms of iron sequestration versus release, we checked the proliferation rate of tumor cells, which were stimulated with supernatants of LPS/IFNγ- or IL-10-treated MΦ (Fig 1E and 1F). We determined mRNA expression of the two known proliferation markers proliferating cell nuclear antigen (PCNA) (Fig 1E) and Ki-67(Fig 1E) [29, 30] as well as PCNA protein expression (Fig 1F), and analyzed proliferation in real-time using the xCELLigence system (Fig 1G). In line with our previous results regarding macrophage iron polarization, we observed a significantly reduced tumor cell proliferation upon incubation of tumor cells with LPS/IFNγ-stimulated MΦ supernatants. However, stimulation of tumor cells with IL-10-treated MΦ supernatants significantly enhanced MCF-7 proliferation. The supplementation of growth medium with FeCl_3_ served as positive control [31].

Taken together, our results indicated that alternatively activated MΦ (IL-10 treatment) show an iron-release phenotype and stimulate tumor cell survival and proliferation, whereas classically activated MΦ (LPS/IFNγ) induce an iron-sequestration phenotype with functional consequences on inhibition of tumor cell proliferation, which is in line with previous reports [6, 32]. As such, we sought to investigate the effect of iron scavengers on the polarization and corresponding phenotype of macrophages, which could therefore impact overall tumor growth.

### Effects of iron chelation in macrophages

**Fig 2:**
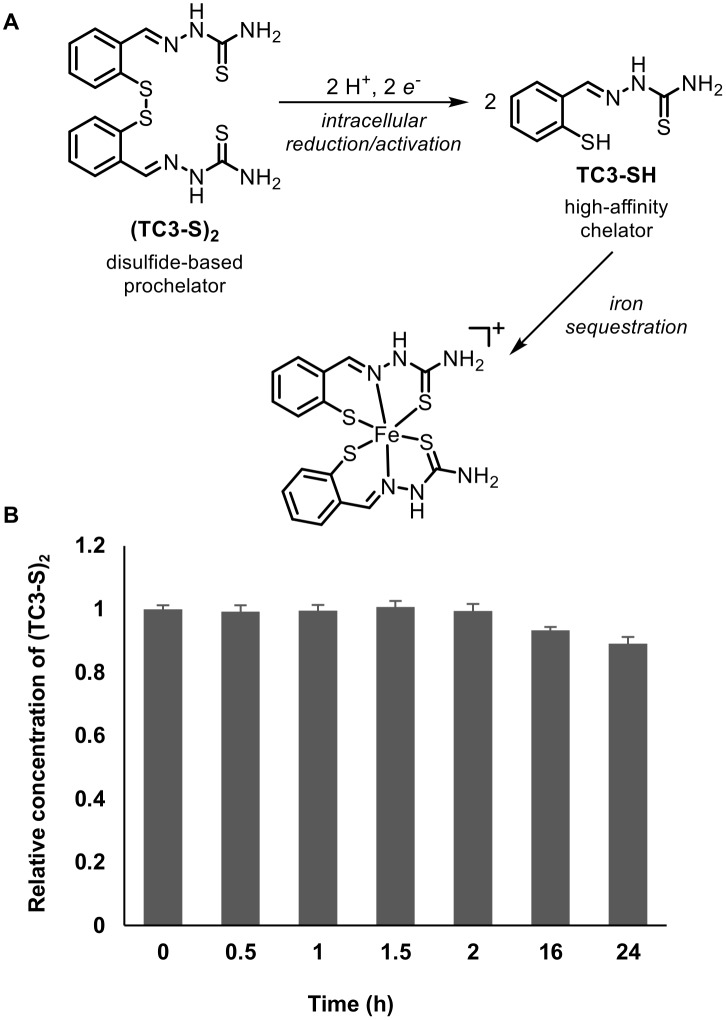
Prochelation approach and stability in cell culture medium. (A) Scheme of the redox directed chelation system: The disulfide-based prochelator (TC3-S)_2_ undergoes intracellular reduction to produce the active chelator TC3-SH, which readily binds iron forming a stable complex. (B) Stability of prochelator (TC3-S)_2_ at 12.0 μM in phenol red-free EMEM: relative concentrations over the course of 24 hours were determined by UV-Visible absorption spectroscopy (λ_max_ = 318 nm, 37°C). No measureable loss of the prochelator is observed over the course of 2 hours, and less than 10% loss is observed over 24 hours.

We analyzed the activity of a recently developed chelation system in primary human MΦ. Prochelator (TC3-S)_2_ (Fig 2A), which features a disulfide linkage between two thiosemicarbazone units, lacks sufficient donor ability and appropriate geometry for iron coordination in aqueous solutions (including growth media). This construct is in turn activated upon cell entry through reduction and disulfide cleavage by intracellular reductants (e.g., glutathione) liberating thiol TC3-SH, which promptly coordinates iron with a high-affinity tridentate binding unit [27, 28]. Particularly relevant to the present study, prochelator (TC3-S)_2_ remains intact in complete growth media and therefore does not interfere with extracellular iron levels (Fig 2B).

**Fig 3:**
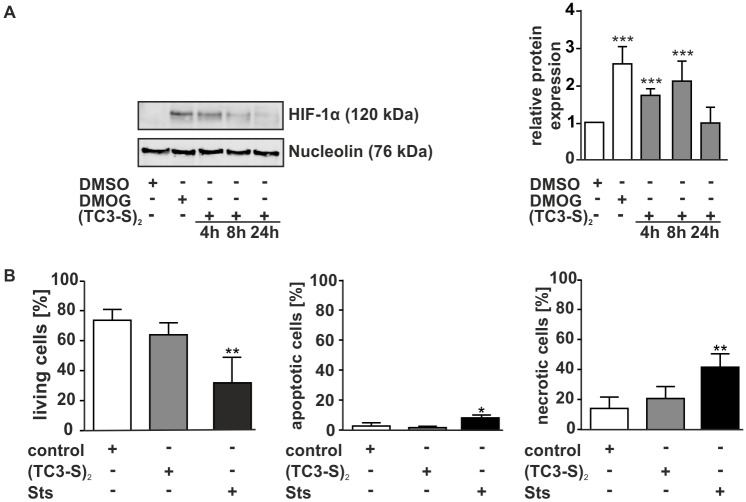
Iron chelator functionality in primary human macrophages. (A) Time dependent activation of HIF-1α in primary human macrophages was determined by Western blot analysis. DMOG- as well as (TC3-SH)_2_-treated cells were compared to DMSO-stimulated control macrophages. (B) Cell viability was measured by AnnexinV/PI staining using FACS measurement. The percentage of living, apoptotic, and necrotic cells is represented. Staurosporine (Sts) was used as a positive control. Data are shown as mean ± S.D.M, n>4, *p<0.05, **p<0.01, ***p<0.001 vs. DMSO.

As a molecular target to test functionality of the chelators, we chose hypoxia-inducible factor-1α (HIF-1α). Because HIF-1α is marked for degradation by iron-dependent prolyl hydroxylases (PHD), iron deprivation results in deactivation of PHD and HIF-1α accumulation [33]. As a positive control, we used the PHD inhibitor dimethyloxalyl glycine (DMOG), which leads to an oxygen-independent and long-lasting activation of HIF-1α [34]. We incubated MΦ in the presence of (TC3-S)_2_ for 4 h, 8 h, and 24 h and measured the accumulation of HIF-1α by Western analysis (Fig 3A). Our results show a significant induction of HIF-1α protein already after 4 h of chelator treatment. (TC3-S)_2_ peaked at a time-point of 8 h and decreased its activity after 24 h, where only a slight induction could be observed. These experiments were critical to establish the timeframe of the subsequent experiments to study macrophage phenotype in the presence of the chelator and we decided to incubate the chelator for 4 h in further experiments. In addition, we performed Annexin V/PI stainings in order to rule out cytotoxic effects of the used chelator in our setting (Fig 3B). As a positive control, we used staurosporine. Results showed that the chelator used in this study did not compromise MΦ viability, which is of critical importance for further experiments using the chelator in combination with polarization treatments.

### Chelator treatment induces a macrophage phenotype shift towards iron-sequestration

**Fig 4:**
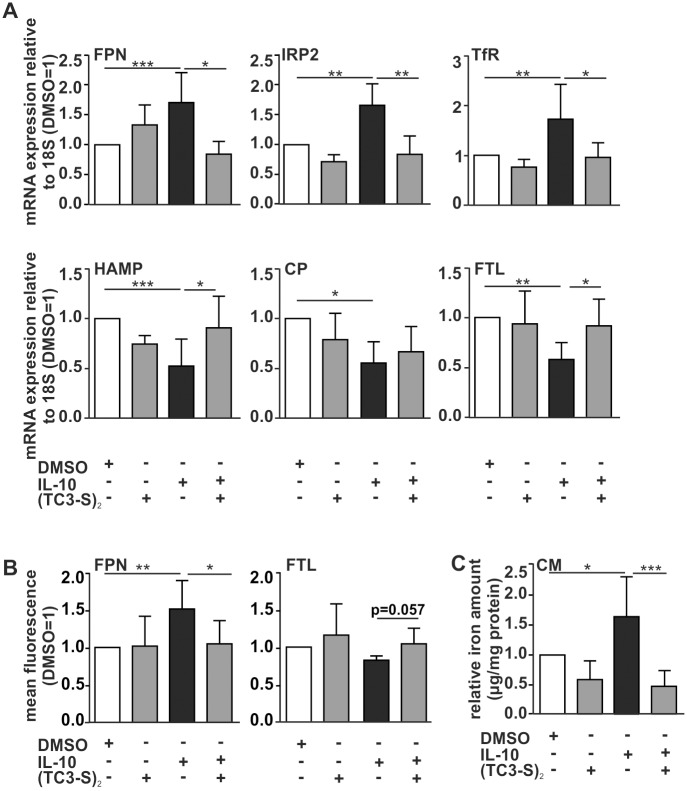
Chelator treatment induces a macrophage phenotype shift towards iron-sequestration. (A) mRNA expression of iron regulated genes FPN, IRP2, TfR, HAMP, CP, and FTL was quantified using qRT-PCR. Protein expression of FPN (B) and FTL (C) was determined by FACS analysis. (D) Iron content in macrophage supernatants was quantified by AAS measurement. Data are shown as means ± S.D.M, n>6, *p<0.05, **p<0.01, ***p<0.001 vs. DMSO.

Having established that the chelator is functional and non-toxic in MΦ, we tested the role of the iron chelator regarding the MΦ iron phenotype in terms of polarization. We incubated MΦ with the chelator for 4 h under unstimulated conditions to define the basal effect on the MΦ phenotype. In order to check the ability to modulate the MΦ iron-release phenotype, we pre-stimulated macrophages for 4 h with IL-10 in order to induce the previously observed iron-release phenotype and treated them for additional 4 h in combination with the chelator for mRNA analysis using qRT-PCR. For protein detection, MΦ were pre-stimulated with IL-10 for 20 h and additional 4 h with experimental chelator. Again, an iron-regulated gene profile was analyzed at mRNA (Fig 4A) and protein level (Fig 4B). No difference was observed in the expression of the iron exporter FPN under basal conditions. However, chelation treatment of IL-10-pre-stimulated MΦ significantly decreased FPN expression. Furthermore, we analyzed the expression of IRP2 and TfR upon chelator treatment. Under basal conditions, both genes were downregulated. However, the IL-10-induced upregulation of both IRP2 and TfR could be reversed by chelator co-treatment. Moreover, we measured the FPN ligand HAMP and the ferroxidase CP, which were both downregulated under basal conditions. HAMP was reduced after IL-10 treatment, but significantly enhanced upon (TC3-S)_2_ co-treatment, whereas CP remained unaffected in IL-10-pre-treated MΦ. The iron storage protein FTL showed no difference under basal conditions, whereas chelation treatment of IL-10-pre-stimulated MΦ significantly enhanced FTL mRNA expression, which was also corroborated at protein level. Again, these results could be confirmed using IL-4 as another M2-stimulus (S2A Fig).

As a functional readout, we measured the cellular iron content in MΦ supernatants by AAS (Fig 4C). We observed no basal effect of chelator treatment in MΦ supernatants compared to the control. However, the significant IL-10-induced increase of iron concentration in the supernatant was reversed upon co-treatment with chelator.

### Functional consequences of chelator-dependent reprogramming of the macrophage iron phenotype in breast cancer cells

**Fig 5:**
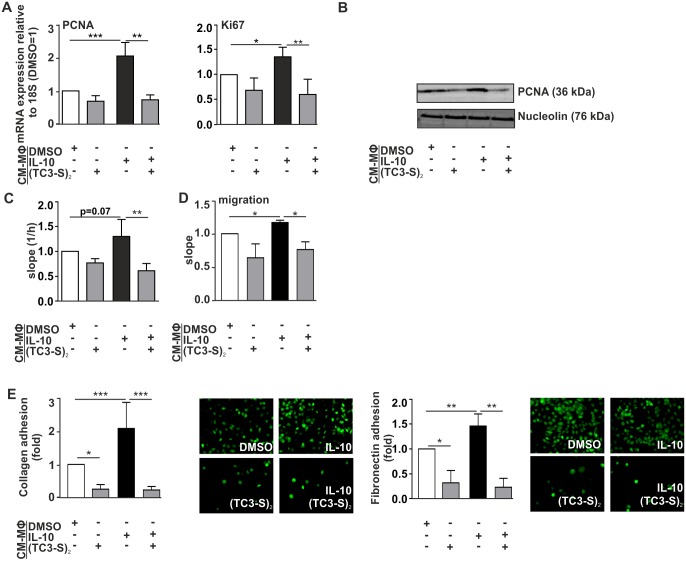
Functional consequences of chelator-dependent reprogramming of the macrophage iron phenotype in breast cancer cells. As a functional readout, proliferation of macrophage conditioned media (CM)-stimulated MCF-7 cells was measured by qRT-PCR of (A) PCNA and Ki-67, (B) Western analysis of PCNA, as well as using (C) the xCELLigence real-time analysis system. (D) Migration and (E) adhesion to either collagen I or fibronectin matrix of MCF-7 tumor cells upon MΦ-conditioned media treatment. (F) Data are shown as means ± S.D.M, n>3, *p<0.05, **p<0.01, ***p<0.001 vs. CM_DMSO.

Since the iron-release phenotype observed after IL-10-stimulation could be blocked by chelator co-treatment, we aimed at determining the functional consequences of the MΦ iron phenotype modulation using the proliferation setting introduced above. To this end, we incubated human MCF-7 breast cancer cells with the supernatant of macrophages treated with either (TC3-S)_2_ alone or in combination with IL-10. The quantification of PCNA (Fig 5A) and Ki-67 mRNA expression (Fig 5B) as well as real-time measurement of cellular proliferation using xCELLigence (Fig 5C) corroborated our previous findings. Incubation of MCF-7 cells with supernatants of MΦ previously treated with chelators did not have any effect on cellular proliferation, whereas supernatant of IL-10-stimulated MΦ significantly enhanced tumor cell proliferation. However, supernatants of IL-10/chelator-treated MΦ significantly reduced the proliferation outcome as compared to IL-10 alone. This observation is consistent with those reported by Recalcati et al. in a different experimental setup employing the cell-impermeant chelator HM-DFO to study the effect of macrophage-conditioned media on cancer cell proliferation [6].

Furthermore, we tested the metastatic potential of MΦ-conditioned media on MCF-7 breast cancer cells. Since migration through the ECM is considered critical during early steps of the metastatic cascade, we examined the effect of macrophage-secreted iron on tumor cell migration using the xCELLigence system (Fig 5D). We observed that conditioned media from M2-polarized, iron-release MΦ (IL-10-treatment) significantly enhanced MCF-7 cancer cell migration, whereas the treatment of tumor cells with supernatants of MΦ previously co-treated with IL-10 and chelator reversed this effect. Moreover, the attachment of tumor cells to extracellular matrix (ECM) components of the lung, including collagen I and fibronectin, is an essential step for tumor cell dissemination. We observed that MΦ-secreted iron after IL-10 treatment significantly enhanced adhesion to both matrices (Fig 5E), whereas prior treatment of MΦ with the chelator significantly blocked this effect.

Taken collectively, our results indicate that treatment with (TC3-S)_2_ is able to reverse the IL-10-induced MΦ iron-release phenotype with functional consequences on cellular iron content, tumor cell proliferation and metastatic behavior.

## Discussion

Cancer cells evolved specialized mechanisms for iron acquisition, storage, and transport in order to ensure their enhanced metabolic turnover. Therefore, tumor-evocated iron handling has emerged as an important aspect of tumor progression. In comparison to healthy cells, cancer cells show enhanced iron sequestration, which results in the development of more aggressive tumors [35–37]. A growing number of studies were conducted to explore the role of iron-related proteins in the context of cancer [6, 7, 38]. The reduced expression of the iron exporter FPN in tumor cells was correlated to the aggressiveness of breast cancer subtypes [23]. High tumor cell FPN levels were correlated to other well-established prognostic markers for better patient survival and outcome, such as the absence of estrogen receptor, low histological grade, and low spread of disease to the lymph nodes. In node-negative breast cancer patients, FTL stored in TAMs was validated as a prognostic biomarker [39]. Because of the growing evidence on their tumor-promoting effects, the expression of iron-regulated genes could become an important factor among the prognostic markers of tumorigenesis.

The role of iron-regulated genes was mainly studied in tumor cells: numerous studies have examined methods to interfere with iron handling in cancer cells, either by directly modulating iron-regulated genes or by the use of iron chelators. Iron chelating agents inhibit DNA synthesis and typically cause a G1-S-phase cell cycle arrest [40], attenuate epithelial-mesenchymal-transition [41], correct the disturbed epigenetic signature of malignant tumor cells [19], and promote cancer cell apoptosis. Nevertheless, a detailed knowledge of the effects on chelators within the tumor microenvironment (and on potential iron sources thereof) is still lacking.

Due to their important role in tumor development and iron handling, we proposed that MΦ might adopt a pro-tumorigenic iron-release phenotype, whereby tumor growth is favored [42]. MΦ are one of the major populations of functionally polarized immune cells in tumors, and their abundance is often associated with a poor patient prognosis [43, 44]. This observation is mainly correlated to their potential to support tumor development at various stages by producing growth and survival factors, by recruiting blood vessels to the tumor (angiogenesis), and supporting metastasis by promoting tumor cell invasion, migration, and intravasation [45]. Intriguingly, because they are central players in systemic iron homeostasis, MΦ have evolved unique mechanisms to recycle, store, and release iron to their local microenvironment. MΦ iron homeostasis is therefore coupled to their functional heterogeneity and plasticity, and the MΦ polarization process dictates expression profiles of genes involved in iron metabolism.

We and others [6, 46] observed that the treatment of MΦ with LPS/IFNγ enhanced the sequestration of iron within the cell, whereas stimulation with anti-inflammatory cytokines such as IL-10 or IL4/IL-13 induced the release of iron from MΦ. This goes in line with a typical cytokine/chemokine profile of M1- and M2-MΦ. However, it is still not exactly defined if the MΦ polarization process dictates intracellular iron handling, or rather if iron availability determines the MΦ phenotype. So far, it is known that pro-inflammatory cytokines such as IL-6 induce the expression of iron-dependent genes in MΦ [47], resulting in iron retention. Moreover, it was also shown that the amount of intracellular iron regulates cytokine formation and polarization of macrophages [48, 49]. Apparently, the control of iron availability is a central step during innate immune responses and thus MΦ are positioned at the interface of immunity and iron homeostasis. In this context, the MΦ iron signature emerges as an additional component of a more complex polarization process to adapt the MΦ phenotype to adverse environments, such as chronic inflammatory disease states, i.e. within tumors.

The presence of immune cells, in particular MΦ, and inflammatory mediators in tumor tissue is a hallmark of chronic inflammation and closely linked to the outcome regarding tumor progression. Therefore, it might be speculated that iron sequestration in MΦ depletes the microenvironment, thereby fostering inflammation and leading to tumor suppression. In contrast, iron release from MΦ would contribute to bystander cell proliferation and survival, which is important for tumor progression [42]. This is particularly relevant since it was appreciated that tumor cells are not able to acquire a fully invasive potential without the recruitment and the support of tumor-infiltrating immune cells, especially MΦ [50–52]. Along this line, recent studies could show that lymphocytes and MΦ synthesize and secrete ferritin which, in turn, stimulates tumor cell proliferation in an iron-dependent manner [39, 53–55]. These data reinforce the importance of the tumor microenvironment in terms of the iron-handling capacity of tumor-infiltrating immune cells, in particular MΦ. Considering that MΦ constitute a major fraction of the infiltrates in most solid tumors and their pivotal role in iron metabolism, the implication of TAMs in iron distribution within the tumor microenvironment and their response to iron chelators represent important areas of investigation in contemporary cancer biology.

In order to assess the impact of iron sequestration on MΦ function, we employed the disulfide-based prochelator (TC3-S)_2_, which is converted to thiol chelator TC3-SH upon intracellular reduction. The prochelator scaffold, namely the species added to growth media in the present studies in cultured cells, is not suitable for iron coordination and therefore this chelation system targets intracellular iron and does not alter iron availability in the extracellular space. As such, this prochelation strategy is particularly advantageous for the study of iron with respect to the crosstalk between TAMs and cancer cells. In accordance to this hypothesis, the current study provides, for the first time, data on the functionality of intracellular prochelators on re-programing the macrophage phenotype from iron release towards iron sequestration with its functional consequences on breast cancer cells. In line with our study on the use of iron chelators in macrophages, Corna et al. previously reported the use of DFO in M2-like bone marrow-derived macrophages [46]. In contrast to our results, they observed an enhanced expression of TfR, whereas we detected a significant decrease in TfR in chelator-treated M2-like primary human macrophage. However, FPN expression was reduced in both experimental settings. These distinct results could be attributed to differences between the mouse and the human system, and/or might be stimulus-dependent (IL-4 vs. IL-10 stimulation). An additional explanation for the observed discrepancy on TfR expression could be the specificity of prochelator system (TC3-S)_2_, which acts exclusively within cells, whereas DFO is also active extracellularly. Furthermore, the different incubation times for the chelators (overnight vs. 4 h in our case) could capture different phases of the cellular response to iron deprivation. Collectively, our findings indicate that disulfide-based prochelators could affect cancer cell proliferation both directly [28] and through the emerging tumor-promoting programs of iron handling by macrophages in the tumor microenvironment. Specifically, we observed that macrophage-secreted iron promotes not only tumor cell growth, but also enhanced the metastatic behavior of cancer cells. In contrast, stimulation of breast cancer cells with supernatants from chelator-treated, re-programmed iron sequestration macrophages significantly blocked this effect. Moreover, previous studies using DFO underscore the importance of macrophage-released iron for tumor cell proliferation and T cell activation [56].

Summarizing, our results support the notion that macrophages and their iron-release phenotype favor tumor progression and metastasis. Future *in vivo* studies should address the possibility to interfere with tumor development by using macrophage-targeted chelation strategies. Moreover, further investigation is needed to define at a molecular level how cancer cells actually manage to acquire iron from their microenvironment and how they manipulate macrophages to serve them as an additional iron source in order to maintain their enhanced metabolism and growth.

## Supporting Information

S1 Fig Polarization profile of IL-10-treated macrophages. (A) Surface expression of polarization markers CD86, CD206, and CD80, measured by FACS analysis. (B) mRNA expression of polarization markers CCL18, CCL2, CD163, TGM2, and TNF-α. Data are shown as means ± S.D.M, n>6, *p<0.05, **p<0.01, ***p<0.001 vs. control.
(TIF)

S2 Fig Chelator treatment of IL-4-treated macrophage induces a phenotype shift towards iron-sequestration. mRNA expression of iron regulated genes FPN, IRP2, TfR, HAMP, CP, and FTL was quantified using qRT-PCR. Data are shown as means ± S.D.M, n>4, *p<0.05, **p<0.01, ***p<0.001 vs. DMSO.
(TIF)
