# Supplementary figures and images for: Intracellular Iron Chelation Modulates the Macrophage Iron Phenotype with Consequences on Tumor Progression
Source: PLoS One. 2016 Nov 2;11(11):e0166164. doi: 10.1371/journal.pone.0166164 (PMC5091876; doi:10.1371/journal.pone.0166164)

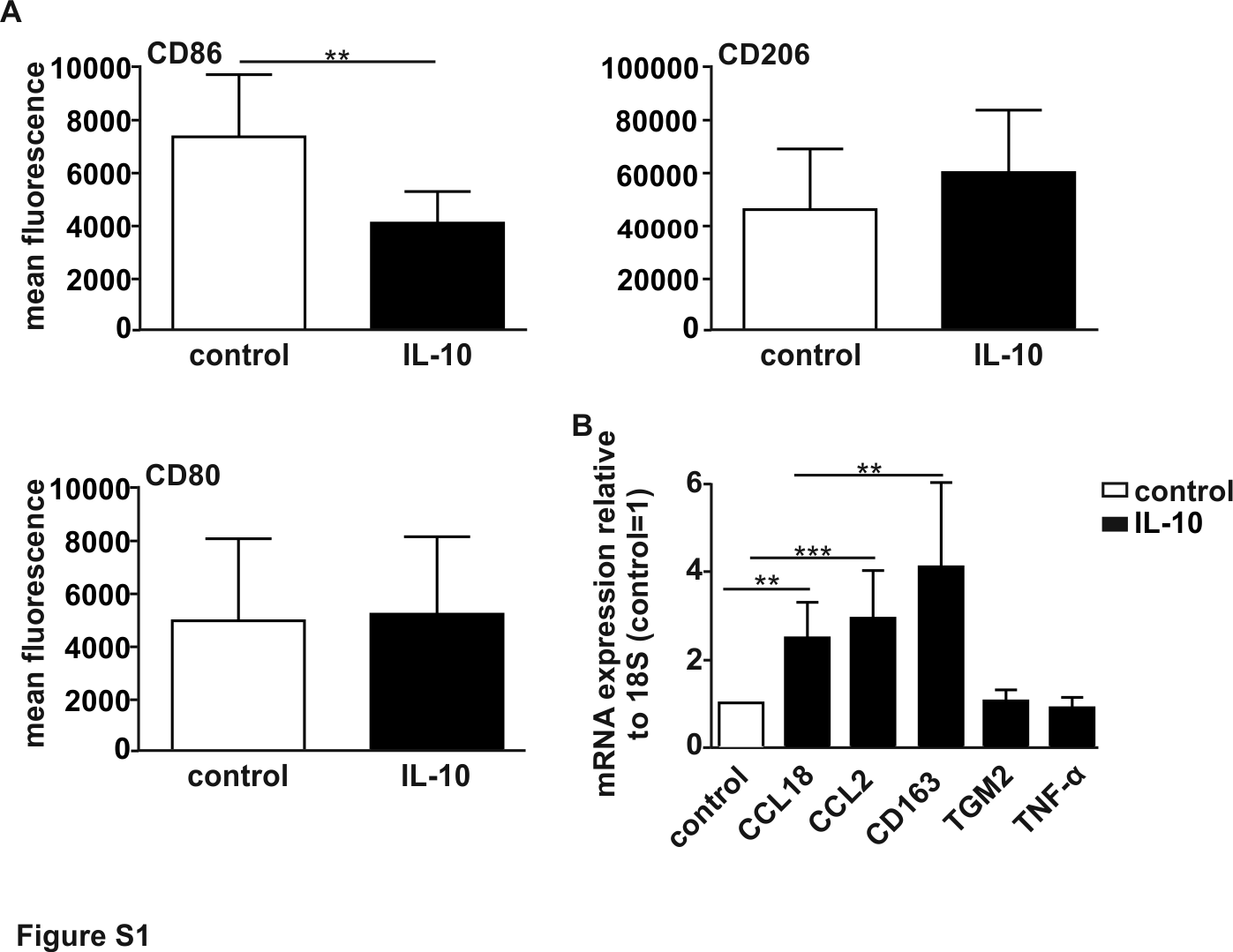

Supplement: S1 Fig — (A) Surface expression of polarization markers CD86, CD206, and CD80, measured by FACS analysis. (B) mRNA expression of polarization markers CCL18, CCL2, CD163, TGM2, and TNF-α. Data are shown as means ± S.D.M, n>6, *p<0.05, **p<0.01, ***p<0.001 vs. control. (TIF) [file pone.0166164.s001.tif]

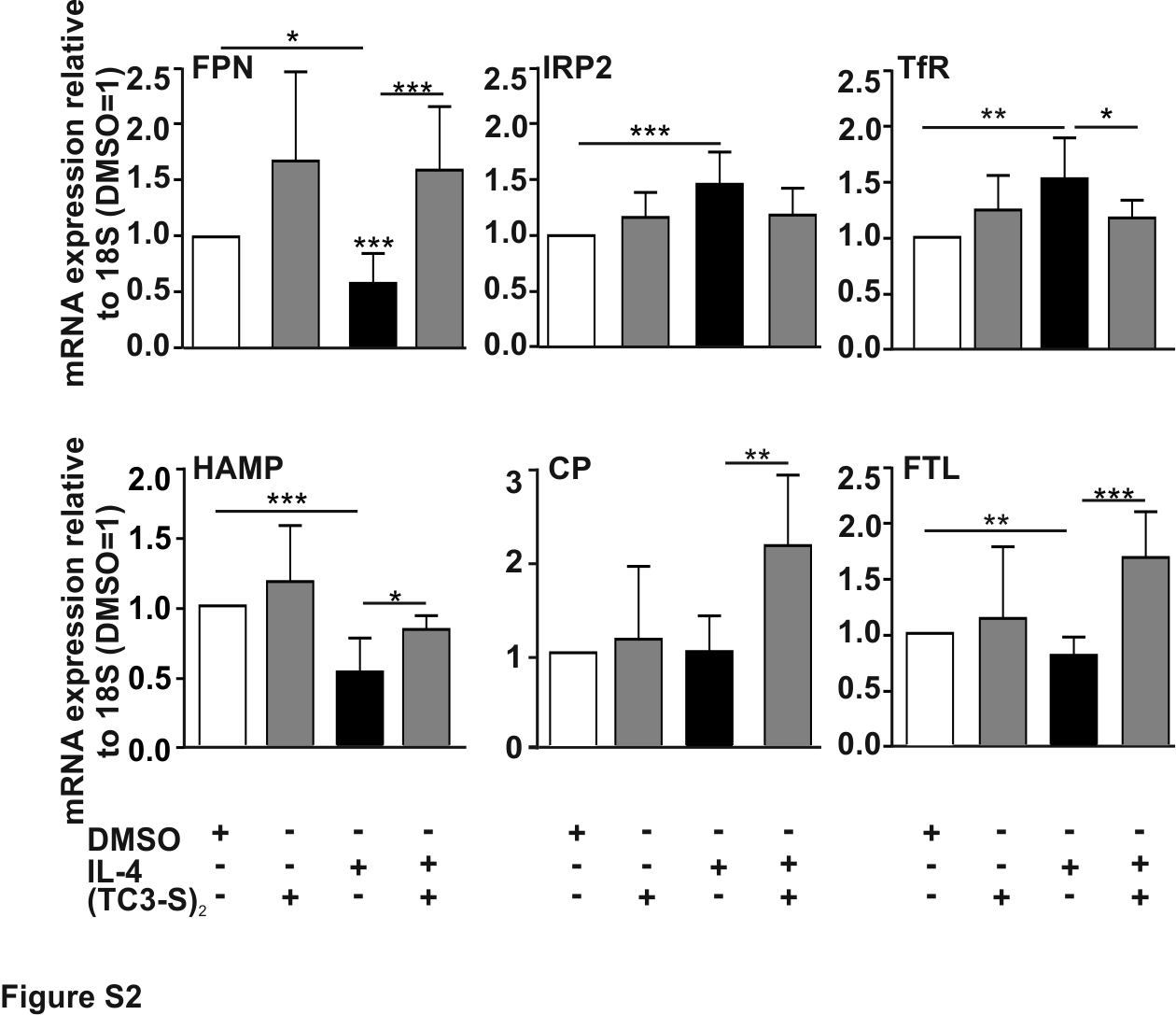

Supplement: S2 Fig — mRNA expression of iron regulated genes FPN, IRP2, TfR, HAMP, CP, and FTL was quantified using qRT-PCR. Data are shown as means ± S.D.M, n>4, *p<0.05, **p<0.01, ***p<0.001 vs. DMSO. (TIF) [file pone.0166164.s002.tif]
